# Delayed Bone Age Might Accelerate the Response to Human Growth Hormone Treatment in Small for Gestational Age Children with Short Stature

**DOI:** 10.1155/2019/8454303

**Published:** 2019-12-18

**Authors:** Jung-Eun Moon, Cheol Woo Ko

**Affiliations:** Department of Pediatrics, School of Medicine, Kyungpook National University, Kyungpook National University Hospital, Daegu, Republic of Korea

## Abstract

**Purpose:**

Growth hormone (GH) treatment is recommended to improve growth and psychosocial problems in short stature children born small for gestational age (SGA). Although GH therapy in these patients has been extensively studied, the impact of therapy according to delays in bone age (BA) is not known well.

**Objective:**

To investigate the effects of GH therapy in SGA patients with short stature according to BA delay.

**Methods:**

We retrospectively analyzed changes in height SD score (SDS) and BA/chronological age (CA) after 6 and 12 months of GH therapy in patients grouped according to BA delay. We studied 27 SGA children with short stature in the pediatric endocrinology clinic of Kyungpook National University Children's Hospital.

**Results:**

Of the 27 patients, 9 had <2 years of BA delay, while 18 had >2 years of delay. There were no significant differences between the two groups in terms of gestational age and weight at birth, height SDS, IGF-1 SDS, and growth hormone dosage at the beginning of therapy. However, height SDS increased significantly in the group with >2 years of BA delay after 6 months of GH therapy (−2.50 ± 0.61 vs −1.87 ± 0.82; *p*=0.037) and 12 months (−2.27 ± 0.70 vs −1.63 ± 0.65; *p*=0.002). When height SDS was compared between with and without GHD, there were no significant differences.

**Conclusions:**

Delayed BA (>2 years) may impact the response to GH treatment in SGA children with short stature.

## 1. Introduction

Small for gestational age (SGA) is defined as a birth weight of <2 SDs according to regional ethnicity references [1–3]. Those who are born SGA have an increased risk of having a short stature [4–6]. Although the majority of SGA patients reach an appropriate size for their age within 2-3 years of birth, approximately 10% do not [7, 8].

Those who do not catch up in growth often have a short stature (height SD score (SDS) <2) during adolescence and adulthood [4, 9], and this is also associated with the development of psychosocial and/or behavioral problems [10]. Therefore, growth hormone (GH) therapy is usually provided to restore normal body height and to improve psychosocial and behavioral problems in SGA children who do not naturally catch up in growth [11–13].

Bone age (BA) is used as an important indicator of biological maturation in children with short stature [14]. Delays in BA are observed in patients with constitutional delay of growth, GH deficiency (GHD), hypothyroidism, malnutrition, and chronic illness [14]. SGA children also present with varying delays in BA, ranging between 1 and 2 years, until puberty [15]; they also have a short final body height because the BA delay is lost without extra growing time after the beginning of puberty [14].

GH therapy has been reported to be more effective when it is initiated before puberty in SGA patients with short stature [16]. Moreover, high doses of GH therapy have been reported to be effective in these patients [13]. Increases in BA have also been reported in SGA patients with short stature who undergo GH therapy [17–19]; although these changes are not associated with age or GH dose [17], bone maturation has been reported to be associated with body height growth [15]. However, no study to date has investigated the effects of GH therapy in association with BA delay in SGA patients with short stature. Therefore, the present study aimed to investigate the influence of GH therapy on BA delay in these patients.

## 2. Materials and Methods

### 2.1. Subjects

Children who visited the pediatric endocrinology clinic at Kyungpook National University Children's Hospital between January 2000 and January 2012 for short stature (defined as height less than two SDs below the mean) and who received more than one year of GH therapy for SGA (full-term infants with gestational age exceeding 37 weeks and body weight for gestational age less than two SDs) were selected for this study (*n* = 27). At the beginning of treatment, all participants were prepubertal according to the Tanner scale in which the start of puberty (stage 1) is defined as breast development for females and a testicular volume <4 ml for males. Patients with chromosomal abnormalities, bone lesions, chronic diseases that cause growth retardation, and a past history of steroid or sex hormone treatment were excluded. Moreover, for all participants, we also evaluated whether GHD influences the effects of GH therapy.

### 2.2. Methods

Patients' medical records were retrospectively analyzed. Details on sex, gestational age, body weight at birth, age at the start of GH therapy, height standard deviation score (SDS) at the start of GH therapy, body weight SDS at the start of treatment, BMI *z*-score at the start of treatment, BA, CA-BA, BA/CA, insulin-like growth factor-1 (IGF-1) SDS, presence of GHD, and GH therapy dosage were collected. Changes in height SDS after 6 and 12 months of GH therapy were compared.

A total of 27 SGA patients who did not have catch-up growth were classified into two groups based on whether they had >2 years or <2 years of BA delay. Data gathered from each group at the start of GH therapy were compared. In addition, changes in height between the two groups after 6 and 12 months were compared. The patients were also classified into two groups based on whether they had GHD; data between the two groups, including changes in height after 6 and 12 months, were compared.

GH stimulation tests were performed in all patients: levodopa- and insulin-induced stimulation testing was conducted, and a human GH level <7 was defined as GHD. For BA analysis, Greulich and Pyle atlas was used by two endocrinologists, and their readings were averaged [20].

### 2.3. Statistical Analysis

Statistical analyses were conducted using SPSS. Comparisons between the two groups were made using paired *t* tests, and repeated measures analysis of variance (ANOVA; Greenhous-G) was used to analyze changes for 1 year after GH treatment. A *p* value < 0.05 was considered to be statistically significant.

## 3. Results

### 3.1. Patient Characteristics

Patient characteristics are given in Table 1. A total of 27 children were investigated in this study, of whom 10 were male (37%) and 17 were female (63%). The mean gestational age was 37.7 ± 2.71 weeks, and the mean body weight at birth was 2239.25 ± 447.0 g. The mean ± SD age at the start of GH treatment was 7.2 ± 1.9 years, height SDS was −2.53 ± 0.44, and the body mass index (BMI) *z*-score was −0.51 ± 0.92. The mean ± SD BA, CA-BA, and BA/CA were 5.0 ± 2.7 years, 2.2 ± 1.5 years, and 0.66 ± 0.24, respectively. The IGF-1 SDS was −1.06 ± 0.86. An average insulin dose of 0.14 ± 0.05 IU/kg/day (0.006 ± 0.002 mg/kg/day) was administered. Among the 27 included SGA patients, 14 (51.9%) were diagnosed with GHD after GH stimulation testing.

### 3.2. Classification Based on the Presence of GHD

Comparing patients with and without GHD (14 vs 13 patients, respectively), there was no significant difference between the two groups in terms of their gestational age (38.5 ± 2.26 vs. 37.0 ± 2.96 months, respectively; *p*=0.154) and body weight at birth (2237.69 ± 500.76 vs. 2240.71 ± 410.14 g, respectively; *p*=0.986). There were also no significant group differences in age, BMI *z*-score, and height SDS at the start of therapy (*p*=0.784, 0.805, and 0.239, respectively). Moreover, CA-BA, BA/CA, IGF-1 SDS, and GH dosage (IU/kg) did not differ significantly between the two groups (*p*=0.117, 0.200, 0.921 and 0.341, respectively) (Table 2).

**Figure 1:**
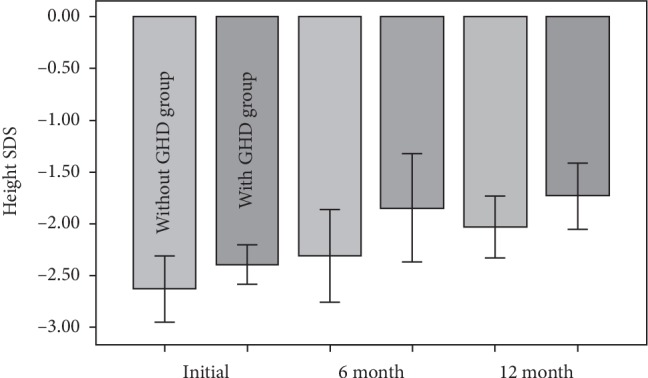
No significant changes in height standard deviation scores (SDS) in the two groups based on whether they had growth hormone deficiency (GHD).

Changes in height SDS, BA/CA, and IGF-1 SDS after 6 and 12 months of GH treatment were analyzed to identify whether there were any significant differences in GH treatment effects in patients with and without GHD. In the group without GHD, there were no significant changes in height SDS at 6 and 12 months (−2.63 ± 0.52 vs −2.30 ± 0.74 at 6 months and −2.02 ± 0.99 at 12 months; *p*=0.085). However, in the group with GHD, the height SDS increased significantly from baseline after 6 and 12 months of treatment (−2.42 ± 0.33 vs −1.87 ± 0.83 at 6 months and −1.73 ± 0.60 at 12 months; *p*=0.001). No significant change in BA/CA was observed in the groups with and without GH (*p*=0.156 and *p*=0.234, respectively). There also was no significant difference in height SDS at 6 and 12 months between the two groups (*p*=0.165 and *p*=0.504, respectively) (Figure 1). Finally, there was no significant difference in IGF-1 SDS between the groups at 6 and 12 months (*p*=0.445 and *p*=0.744, respectively).

### 3.3. Classification Based on Two Years of Delay in BA

Among a total of 27 patients, 9 had <2 years of BA delay and 18 had >2 years of delay. There was no significant difference between the two groups in terms of gestational age and weight at birth (38 ± 2.2 vs 37.6 ± 3.0 months and 2234.4 ± 374.9 vs 2241.7 ± 489.4 g, respectively; *p*=0.726 and *p*=0.969). The group with less than 2 years of BA delay tended to be younger at the start of GH therapy (8.1 ± 1.6 vs 6.7 ± 1.9 years, respectively; *p*=0.061). The groups did not differ significantly in terms of BMI, height SDS, IGF-1 SDS, and GH dose (IU/kg) at the beginning of the therapy (Table 3).

**Figure 2:**
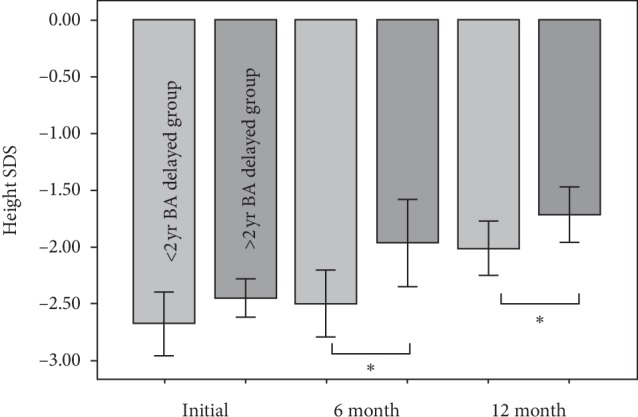
Significant changes in height standard deviation scores (SDS) at the 6-month and 12-month follow-up in the two groups based on bone age delay ^*∗*^*p* < 0.05.

We also evaluated whether there were any treatment differences at 6 and 12 months between the two groups by assessing changes in height SDS, BA/CA, and IGF-1 at these timepoints. No significant differences were observed for height SDS at 6 and 12 months in the group with <2 years of BA delay. In the group with >2 years of BA delay, height SDS at 6 and 12 months increased significantly from baseline (from −2.45 ± 0.34 to −1.87 ± 0.82 at 6 months and −1.63 ± 0.65 at 12 months; *p*=0.01 and 0.001, respectively). However, no significant differences in BA/CA were observed in either group. The group with >2 years of BA delay had significant increases in height SDS at 6 and 12 months when compared to the group with more than 1 year of BA delay (−2.5 ± 0.61 vs −1.87 ± 0.82 at 6 months, −2.27 ± 0.7 vs −1.63 ± 0.65 at 12 months; *p*=0.037 and 0.002, respectively) (Figure 2). IGF-1 SDS did not differ significantly between the groups.

## 4. Discussion

This study is the first to describe the effects of GH treatment according to the degree of BA delay in SGA patients with short stature. BA readings are the first step in diagnosing growth-related diseases in patients with short stature, and they are particularly useful in SGA patients who may have 1-2 years of BA delay before the age of 8 [15, 17]. Moreover, SGA patients whose height is below two SDs after the age of 3 are classified as having SGA without catch-up growth; these patients are also likely to be short when they reach adulthood [14]. In the present study, SGA patients were 7.2 ± 1.9 years of age at the start of treatment, had 2.2 ± 1.5 years of BA delay, and did not have catch-up growth, with a mean height SDS of −2.53 ± 0.44.

In addition to SGA, BA delay is also observed in patients with constitutional delay of puberty (CDP), GHD, hypothyroidism, malnutrition, and chronic illness [14]. Since the patients included in the present study had more than one year of bone delay with short stature, the causes of their short stature were investigated using GH stimulation tests. All patients had normal thyroid function without malnutrition or chronic illness. GHD was observed in 51.9% of the participants. Van et al. reported that the presence of GHD has no effect on treatment outcomes in SGA patients [11]. Our results are in accordance with this conclusion, as the presence of GHD did not impact on height SDS after GH therapy in our cohort.

GH therapy is necessary in short stature with SGA patients without catch-up growth. GH has been reported to influence not only physical growth but also IQ and behavior; therefore, the importance of GH therapy in SGA patients with short stature is evident [10–14]. Houk and Lee reported that GH treatment in SGA patients leads to greater growth during puberty if the treatment starts at younger ages, because growth velocity is greatest during the first 2 years [16]. De Ridder et al. also reported that younger ages at the start of GH therapy are associated with significant increases in patients' final height in adulthood [21, 22]. However, in our study, no significant correlation between age at the start of therapy and changes in height SDS was found (*r* = −0.066, *p*=0.744). This seems to be because most patients were of similar ages, with a mean age of 7.2 ± 1.9.

In a study investigating BA, Arends et al. reported that SGA patients who were not administered GH therapy had persistent BA and growth delays during the three year follow-up period, but those who were administered GH therapy had significant increases in BA and growth during the treatment period [15]. Darendeliler et al. reported that the CA and BA after one year of GH treatment in SGA patients was 2.0 (range, 0.7–3.6) and 1.8 (range, 0.1–3.3) and that the change in height SDS was 0.5 (range, −0.2–1.0); this indicates that increases in BA after treatment comprise normal progression [17]. The BA/CA after one year of GH therapy in GHD and SGA patients has also been reported to vary between 0.7 and 1.5 [11, 23, 24]. Such findings indicate that increases in height SDS resulting from GH therapy do not result from excessive progression of BA caused by GH treatment. In this study, patients' CA-BA changed from 2.2 ± 1.5 years to 2.2 ± 1.9 years after one year of treatment, indicating no significant progression in BA. Similarly, BA/CA changed from 0.65 ± 0.24 to 0.67 ± 0.25 (*p*=0.811), and height SDS changed from −2.5 ± 0.43 to −1.8 ± 0.70 (*p*=0.003); in other words, height SDS increased significantly without a corresponding increase in BA/CA. In comparative analysis between groups, BA/CA changed from 0.51 ± 0.14 to 0.50 ± 0.23 in the group with >2 years of BA delay; however, no significant progression in BA was observed after one year of GH treatment (*p*=0.897). The height SDS increased significantly from −2.45 ± 0.34 to −1.63 ± 0.65 (*p*=0.001). This indicates that significant growth observed after one year of GH treatment in SGA patients with >2 years of BA delay does not result from overprogression of bone aging. According to the present study, SGA patients with short stature and more than 2 years of delay in initial BA can be expected to benefit from GH treatment.

A limitation of this study is that it was conducted on a small number of patients over a relatively short period of observation.

In conclusion, SGA patients with short stature with more than two years of BA delay had better GH therapy effects than did those with less than 2 years of BA delay. Future large-scale, long-term studies should be conducted.

## Figures and Tables

**Table 1:** Clinical and laboratory characteristics of study patients.

Patient no.	Total	Males	Females	*p* value
	27	10	17	
Gestational age (months)	37.7 ± 2.7	37.3 ± 3.7	38.0 ± 2.0	0.208
Birth weight (g)	2239.3 ± 447.0	2298.0 ± 470.5	2204.7 ± 443.6	0.683
Age (years)	7.2 ± 1.9	7.3 ± 2.0	7.1 ± 2.0	0.862
BMI (kg/m^2^, *Z*-score)	−0.51 ± 0.92	−0.78 ± 0.89	−0.35 ± 0.93	0.988
Height SDS	−2.53 ± 0.44	−2.52 ± 0.30	−2.53 ± 0.51	0.349
BA (years)	5.0 ± 2.7	3.8 ± 2.1	5.6 ± 2.9	0.160
CA-BA (years)	2.2 ± 1.5	3.4 ± 1.0	1.4 ± 1.3	0.080
BA/CA	0.66 ± 0.24	0.50 ± 0.16	0.75 ± 0.23	0.110
IGF-1 SDS	−1.06 ± 0.86	−1.21 ± 0.83	−0.96 ± 0.89	0.596
rhGH (IU/kg/day)	0.14 ± 0.05	0.14 ± 0.03	0.16 ± 0.04	0.434
GHD (no., %)	14 (51.9%)	7 (70%)	7 (41.2%)	

No.: number; BA, bone age; CA-BA, differences between bone age and chronological age; BA/CA, bone age to chronological age ratio; rhGH, recombinant human growth hormone; GHD, growth hormone deficiency.

**Table 2:** Initial clinical and laboratory characteristics when patients were classified according to the presence of growth hormone deficiency (GHD).

M : F	Without GHD group (*N* = 13)	With GHD group (*N* = 14)	*p* value
	3 : 10	7 : 7	
Gestational age (months)	38.5 ± 2.26	37.0 ± 2.96	0.154
Birth weight (g)	2237.69 ± 500.76	2240.71 ± 410.14	0.986
Age (years)	7.05 ± 1.85	7.26 ± 1.94	0.784
BMI (kg/m^2^, *z*-score)	−0.46 ± 0.85	−0.55 ± 1.01	0.805
Height SDS	−2.63 ± 0.25	−2.42 ± 0.33	0.239
BA (years)	5.3 ± 2.7	4.6 ± 2.8	0.534
CA-BA (years)	1.71 ± 1.28	2.61 ± 1.59	0.117
BA/CA	0.72 ± 0.23	0.60 ± 0.24	0.200
IGF-1 SDS	−1.03 ± 0.9	−1.07 ± 0.86	0.921
rhGH (IU/kg/day)	0.16 ± 0.03	0.15 ± 0.04	0.341

*N*, number; M : F, male: female (number); BA, bone age; CA-BA, differences between bone age and chronological age; BA/CA, bone age to chronological age ratio; rhGH, recombinant human growth hormone; GHD, growth hormone deficiency.

**Table 3:** Initial clinical and laboratory characteristics when classified according to whether patients had bone age (BA) delay of more or less than 2 years.

	<2 yr BA delayed group (*N* = 9)	>2 yr BA delayed group (*N* = 18)
M : F	0 : 9	10 : 8	*p* value
Gestational age (months)	38.0 ± 2.2	37.6 ± 3.0	0.726
Birth weight (g)	2234.4 ± 374.9	2241.7 ± 489.4	0.969
Age (years)	8.1 ± 1.6	6.7 ± 1.9	0.061
BMI (kg/m^2^, *Z* score)	−0.46 ± 0.86	−0.53 ± 0.97	0.844
Height SDS	−2.67 ± 0.58	−2.45 ± 0.34	0.210
BA (years)	7.6 ± 2.1	3.6 ± 1.9	0.001
CA-BA (years)	0.5 ± 0.9	3.0 ± 0.9	0.001
BA/CA	0.93 ± 0.12	0.51 ± 0.14	0.001
IGF-1 SDS	−1.07 ± 0.77	−1 ± 0.87	0.842
rhGH (IU/kg/day)	0.17 ± 0.04	0.14 ± 0.02	0.074

*N*, number; M : F, male: female (number); BA, bone age; CA-BA, differences between bone age and chronological age; BA/CA, bone age to chronological age ratio; rhGH, recombinant human growth hormone; GHD, growth hormone deficiency.

## Data Availability

The data used to support the findings of this study are included within the article.
